# Association Between Rotavirus Vaccination and Antibiotic Prescribing Among Commercially Insured US Children, 2007–2018

**DOI:** 10.1093/ofid/ofac276

**Published:** 2022-06-09

**Authors:** Eric W Hall, Ashley Tippett, Scott Fridkin, Evan J Anderson, Ben Lopman, David Benkeser, Julia M Baker

**Affiliations:** School of Public Health, Oregon Health and Science University, Portland, Oregon, USA; Department of Pediatrics, Emory University School of Medicine, Atlanta, Georgia, USA; Department of Medicine, Emory University School of Medicine, Atlanta, Georgia, USA; Department of Pediatrics, Emory University School of Medicine, Atlanta, Georgia, USA; Department of Medicine, Emory University School of Medicine, Atlanta, Georgia, USA; Center for Childhood Infections and Vaccines, Children’s Healthcare of Atlanta, Emory University, Atlanta, Georgia, USA; Department of Epidemiology, Rollins School of Public Health, Emory University, Atlanta, Georgia, USA; Department of Biostatistics and Bioinformatics, Rollins School of Public Health, Emory University, Atlanta, Georgia, USA; Department of Epidemiology, Rollins School of Public Health, Emory University, Atlanta, Georgia, USA

**Keywords:** antibacterial agents, prescriptions, rotavirus, vaccination

## Abstract

**Background:**

Vaccines may play a role in controlling the spread of antibiotic resistance. However, it is unknown if rotavirus vaccination affects antibiotic use in the United States (US).

**Methods:**

Using data from the IBM MarketScan Commercial Database, we conducted a retrospective cohort of US children born between 2007 and 2018 who were continuously enrolled for the first 8 months of life (N = 2 136 136). We followed children through 5 years of age and compared children who completed a full rotavirus vaccination series by 8 months of age to children who had not received any doses of rotavirus vaccination. We evaluated antibiotic prescriptions associated with an acute gastroenteritis (AGE) diagnosis and defined the switching of antibiotics as the prescription of a second, different antibiotic within 28 days. Using a stratified Kaplan-Meier approach, we estimated the cumulative incidence for each study group, adjusted for receipt of pneumococcal conjugate vaccine, provider type, and urban/rural status.

**Results:**

Overall, 0.8% (n = 17 318) of participants received an antibiotic prescription following an AGE diagnosis. The 5-year adjusted relative cumulative incidence of antibiotic prescription following an AGE diagnosis was 0.793 (95% confidence interval [CI], .761–.827) among children with complete rotavirus vaccination compared to children without rotavirus vaccination. Additionally, children with complete vaccination were less likely to switch antibiotics (0.808 [95% CI, .743–.887]). Rotavirus vaccination has averted an estimated 67 045 (95% CI, 53 729–80 664) antibiotic prescriptions nationally among children born between 2007 and 2018.

**Conclusions:**

These results demonstrate that rotavirus vaccines reduce antibiotic prescribing for AGE, which could help reduce the growth of antibiotic resistance.

## Teaser

In this retrospective study of commercially insured children, vaccination against rotavirus appeared to reduce the inappropriate prescribing of antibiotics for acute gastroenteritis and subsequently may contribute to reduced antibiotic resistance among children.

Since its introduction in 2006 [1], rotavirus vaccination has had a beneficial impact on child health in the United States (US). Prior to vaccine introduction, rotavirus was the leading cause of severe diarrheal disease among US children <5 years of age [2, 3]. The primary benefit of rotavirus vaccination is the reduction of severe gastroenteritis. Vaccine effectiveness is >85% against severe rotavirus gastroenteritis, and the introduction of vaccination in children has led to a dramatic reduction in rotavirus hospitalizations in all ages and resulting medical costs [4–9]. Additionally, rotavirus vaccination may lead to several nontargeted benefits, such as the reduction of type 1 diabetes and febrile seizures, both of which are potential downstream outcomes of rotavirus infection [10–12].

Vaccines may be an important tool in addressing the growing threat of antimicrobial resistance [13, 14]. In 2019, the Centers for Disease Control and Prevention estimated that >2.8 million antibiotic-resistant infections occur in the US each year, resulting in >35 000 deaths [15]. As part of a comprehensive federal strategy, the Federal Task Force on Combating Antibiotic-Resistant Bacteria identified the use of existing and new vaccines as a key tool for reducing unnecessary antibiotics [16]. There is strong evidence that vaccines targeting bacterial pathogens, such as the *Haemophilus influenzae* type b and pneumococcal conjugate (PCV) vaccines, have resulted in reduced bacterial colonization, subsequent antibiotic use, and prevalence of antibiotic-resistant strains on a population level [13]. Vaccines targeting viral pathogens, such as influenza vaccines, have also shown the potential to reduce unnecessary antibiotic prescribing [13].

Similarly, rotavirus vaccination may impact antibiotic prescribing and resistance by 2 mechanisms. First, a reduction in acute gastroenteritis (AGE) cases could result in fewer inappropriately prescribed antibiotics [13]. In 2010–2011, there were 7.7 million visits by children <19 years of age for AGE in the US and 10.4% of them received an antibiotic (equating to 800 000 antibiotic prescriptions) [17]. When bacterial agents are the cause of gastroenteritis, appropriate treatment sometimes includes antibiotics. However, many enteropathogens, including rotavirus, present with nonspecific symptoms and are often not known to be the etiologic agent unless testing is conducted. Since etiology is usually not known at the time of a medical encounter, antibiotics are frequently prescribed for viral gastroenteritis even though they are not recommended [18]. Second, antibiotics may disrupt the enteric microbiome, which can lead to secondary bacterial infections, which can inherently be resistant to treatment and possibly require microbiome restorative treatments [19, 20].

Therefore, we hypothesized that by preventing viral gastroenteritis, rotavirus vaccination may be associated with less antibiotic prescribing among children in the US. This question has only been investigated in low- and middle-income countries [21, 22], where antibiotic treatment practices are likely different from the US. We used national health insurance claims data to investigate if rotavirus vaccination is associated with a reduction in antibiotic prescribing and the switching of antibiotics among children <5 years of age in the postvaccine era (2007–2018).

## METHODS

### Data Source

Data for our analysis came from the IBM MarketScan Commercial Database [23]. The database contains de-identified, individual-level records on employees, their spouses, and their dependents who have employer-sponsored health insurance from all US states. The database includes data on enrollment status, inpatient and outpatient medical visits, and pharmaceutical claims for several million individuals each year.

### Construction of Cohort

We constructed a retrospective cohort of commercially insured US children who were born between 2007 and 2018. All children whose births were included in MarketScan were eligible to be enrolled. We estimated each individual patient’s date of birth by restricting to *International Classification of Diseases, Ninth Revision* and *Tenth Revision* (*ICD-9/ICD-10*) codes for live births, major diagnostic codes identifying “newborns and other neonates with conditions originating in perinatal period,” and admission codes indicating maternity or newborn admissions. Inpatient and outpatient records were then combined in chronological order, with the earliest live birth claim date used as a proxy for date of birth. Since our primary exposure of interest (rotavirus vaccination) was introduced in the US in 2006 [1], we limited the analysis to children born in 2007 or later. To reduce misclassification of rotavirus vaccination status, we limited the analysis to children who were continuously followed in the dataset for the first 8 months of life (ie, still enrolled 7 months after the birth month) and were born in a state that did not include a universal vaccination purchasing program. We excluded children whose enrollment lapsed during the first 8 months of life as they could have received rotavirus vaccination during that lapse and their vaccination data would not be included in this dataset. Similarly, we excluded children born in states that provide universal vaccination programs (ie, Alaska, Connecticut, Idaho, Massachusetts, Maine, North Dakota, New Hampshire, New Mexico, Oregon, Rhode Island, South Dakota, Vermont, Washington, Wisconsin, and Wyoming) that offer free immunization, which may not be recorded in insurance data.

### Exposure, Outcomes, and Potential Confounders

We used *Current Procedural Terminology* codes 90680 and 90681 to identify all inpatient or outpatient visits that involved receipt of pentavalent rotavirus vaccine or monovalent rotavirus vaccine, respectively. In line with current clinical recommendations [24], children who completed a full rotavirus vaccination series (3 doses of pentavalent rotavirus vaccine or 2 doses of monovalent rotavirus vaccine) by 8 months of age (defined as first 244 days of life) were considered to have complete vaccination (Table 1). In contrast, children who did not receive any rotavirus vaccine by 8 months of age were considered to have no rotavirus vaccination.

**Table 1.** Definition of Variables Used in Analysis of IBM MarketScan Commercial Database

Code Source and Variable	Codes	Definition
*Current Procedural Terminology*		
Complete rotavirus vaccination (exposure)	90680, 90681	Complete series (2 doses of 90681 or 3 doses of 90680) by 8 months of age
No rotavirus vaccination	Same as above	No doses by 8 months of age
PCV vaccination	90669, 90670	At least 2 doses by 8 months of age
*International Classification of Diseases*		
AGE diagnosis	*ICD-9*: 001.0, 001.1, 001.9, 002.0-002.3, 002.9, 003.0, 003.1, 003.8, 003.9, 004.0–004.3, 004.8, 004.9, 005.0–005.4, 005.81, 005.89, 005.9, 006.0–006.2, 006.8, 006.9, 007.0–007.5, 007.8, 007.9, 008.0, 008.00–008.04, 008.09, 008.1–008.4, 008.41–008.47, 008.49, 008.5, 008.6, 008.61–008.67, 008.69, 008.8, 009.0–009.3, 558.9, 787.91 *ICD-10*: A00.0, A00.1, A00.9, A01.0–A01.4, A02.0, A02.1, A02.8–A03.3, A03.8, A03.9, A05.0–A05.3, A05.5, A05.8, A05.9, A06.0–A06.2, A06.8, A06.9, A07.0–A07.2, A07.4, A07.8, A07.9, A04.0–A04.9, A08.0–A08.2, A08.19, A08.31, A08.32, A08.3, A08.39, A09, K52.89, R19.7	Any AGE code after 8 months of age
Therapeutic Class Codes		
Any antibiotic prescription	4 = aminoglycosides, 6 = cephalosporin, 7 = β-lactam, 9 = erythromycin and macrolide, 10 = penicillins, 12 = miscellaneous antibiotics, 16 = quinolones, 17 = sulfonamides and combinations, 18 = sulfones, 19 = urinary anti-infectives, 20 = miscellaneous anti-infectives	Any antibiotic prescription after 8 months of age
Antibiotic prescription following AGE (primary outcome)	Same as above	Any prescription after 8 months of age and within 3 days of an outpatient AGE diagnosis or the discharge date of an inpatient AGE diagnosis
Second antibiotic prescription following AGE	Same as above	National Drug Code number (label and product segment) that is different from first antibiotic prescription and within 28 days of initial prescription that followed an AGE diagnosis

Abbreviations: AGE, acute gastroenteritis; *ICD-9*, *International Classification of Diseases, Ninth Revision*; *ICD-10*, *International Classification of Diseases, Tenth Revision*; PCV, pneumococcal conjugate vaccine.

The primary outcome of interest was an antibiotic prescription associated with an AGE diagnosis before 5 years of age. Based on clinical expert opinion (coauthors E. J. A. and S. F.), we defined a set of antibiotic and anti-infective agents that would likely be prescribed for AGE (Table 1) and extracted the data of the relevant pharmaceutical claims. In parallel, we identified all AGE diagnoses using *ICD-9/ICD-10* codes from inpatient and outpatient visits. An AGE diagnosis was defined as the presence of any AGE diagnostic code after 8 months of age. Any antibiotic prescription that occurred within 3 days of an outpatient AGE diagnostic code or within 3 days of the discharge date from an inpatient visit that included an AGE diagnostic code was considered an antibiotic prescription following AGE. As a secondary outcome, we evaluated the switching of antibiotics as a marker of potential antibiotic resistance. We defined the switching of antibiotics by identifying the prescription of a second, different antibiotic (ie different National Drug Code label and product number within any of the defined therapeutic drug classes) within 28 days of the initial prescription.

Data on several potential confounders, previously identified in the literature, were extracted for each child in the cohort including PCV vaccination status, provider type, and rural/urban location of residence. PCV vaccination status was of interest for several reasons. First, PCV is a routine childhood immunization with high coverage [25] and was, therefore, used to represent healthcare-seeking behavior. Second, PCV is a relatively new vaccine [26] (originally introduced in 2000) and may represent a parent/guardian’s willingness to accept new vaccines such as the rotavirus vaccine. Last, PCV vaccination directly impacts a child’s likelihood of pneumococcal bacterial infection and resulting antibiotic treatment, potentially confounding the rotavirus vaccination and antibiotic prescription relationship. Children who had received at least 2 doses of PCV by 8 months of age were considered to have been vaccinated against PCV for our analysis. Rates of rotavirus vaccination and antibiotic prescribing are known to vary by provider type [27, 28]. We categorized each child into 3 mutually exclusive provider-type categories based on data from outpatient visits. Provider type was categorized as “pediatrician” if a child had any outpatient visits to a pediatrician during the follow-up period. Otherwise, provider type was categorized as “family practitioner” if any of the outpatient visits were to a family practitioner and “other” if the child did not visit a pediatrician or a family practitioner. Last, individuals were categorized as living in a rural area if the primary address for the plan holder was not within one of the defined metropolitan statistical areas [29], in an effort to capture some of the structural factors that may lead to differences in access to healthcare and health education.

### Statistical Analysis

We summarized and compared covariates and outcomes among children who had complete rotavirus vaccination and children who had no rotavirus vaccination. Additionally, we conducted a time-to-event analysis to estimate the unadjusted and adjusted cumulative incidence of antibiotic prescription associated with AGE among children with complete rotavirus vaccination and children with no rotavirus vaccination. Follow-up began at 8 months of age and if children lost coverage before experiencing the outcome, they were censored at the first day of the month in which coverage was lost. Participants who did not experience the outcome during follow-up were censored after 5 years of follow-up.

Within each group, we defined 12 strata based on receipt of PCV vaccine (2 categories), provider type (3 categories), and urban/rural (2 categories). For each group, we estimated the Kaplan-Meier survival probability for each day of follow-up and multiplied it by the proportion of each group within each stratum. The resulting probabilities were summed to generate cumulative incidence curves adjusted for receipt of PCV vaccine, provider type, and urban/rural. Cumulative incidence ratios were estimated by comparing the daily cumulative incidence among children with complete rotavirus vaccination and children without rotavirus vaccination. Confidence intervals (CIs) were estimated using 1000 bootstrapped iterations.

We conducted several sensitivity analyses using alternative outcome definitions. First, we conducted a sensitivity analysis using any antibiotic prescription (with or without an AGE diagnosis), a less specific outcome. Second, we evaluated a more specific outcome definition by conducting a sensitivity analysis that limited the outcome to antibiotic prescriptions with an AGE diagnosis that occurred during the historic rotavirus season (January–June) [30]. Third, we conducted the same stratified time-to-event analysis using the secondary outcome, switching antibiotics following an AGE diagnosis. Finally, we conducted a sensitivity analysis in which we expanded the definition of an AGE event to including *ICD* codes for vomiting [31] (alone and with unspecified nausea; results presented in Supplementary Data).

We extended the primary results to the entire cohort of children born in the US between 2007 and 2018 to estimate a lower bound of the number of antibiotic prescriptions that have been averted by rotavirus vaccination. First, we estimated the number of children who have been vaccinated against rotavirus from 2007 to 2018 using annual birth data from vital statistics [32] and annual estimates of rotavirus vaccination coverage [25, 33]. Next, we multiplied the total number of children vaccinated against rotavirus by the proportion of children with an antibiotic prescription following AGE in our study sample to estimate the total number of vaccinated children who have received an antibiotic prescription following an AGE diagnosis nationally. We divided that by the cumulative incidence ratio and calculated the difference between the 2 quantities to estimate the number of antibiotic prescriptions associated with AGE that have been averted. Data management was done in SAS version 9.4, and all analysis was performed in R version 3.6.3 software.

## RESULTS

From 2007 to 2018, there were 3 739 073 children born in the MarketScan database. We excluded 486 895 (13.0%) children who were born in states with universal vaccination and 1 116 042 (29.8%) children who were not continuously enrolled for 8 months, resulting in an analytic dataset of 2 136 136 children. The median length of follow-up was 993 days (interquartile range, 513–1826 days). Among participants, there were 1 493 605 (69.9%) children with complete rotavirus vaccination by 8 months of age and 336 434 (15.7%) children with no rotavirus vaccination by 8 months of age (Table 2). Children with complete rotavirus vaccination were more likely to have also received a PCV vaccine (99.5%) compared to children without rotavirus vaccination (57.8%). Additionally, participants with rotavirus vaccination were more likely to have seen a pediatrician (88.4%) and less likely to live in a rural area (16.4%) compared to participants without rotavirus vaccination (72.4% and 22.5%, respectively).

**Table 2.** Characteristics of Analytic Cohort^a^, by Rotavirus Vaccination Status, 2007–2018

Characteristic^b^	Total		Complete Rotavirus Vaccination^c^		No Rotavirus Vaccination^c^	
	(N = 2 136 136)		(n = 1 493 605)		(n = 336 434)	
Any antibiotic prescription	1 183 658	(55.4)	846 883	(56.7)	175 103	(52.0)
AGE diagnoses	428 112	(20.0)	295 273	(19.8)	71 252	(21.2)
Antibiotic prescription following AGE	17 318	(0.8)	11 572	(0.8)	3149	(0.9)
Typical rotavirus season (January–June)	9023	(0.4)	5879	(0.4)	1823	(0.5)
Second antibiotic within 28 days	2692	(0.1)	1832	(0.1)	479	(0.2)
PCV vaccination	1 952 983	(91.4)	1 486 065	(99.5)	194 426	(57.8)
Provider type						
Pediatrician	1 818 962	(85.2)	1 319 794	(88.4)	243 396	(72.3)
Family practitioner (no pediatrician)	128 119	(6.0)	60 113	(4.0)	46 209	(13.7)
Other	189 055	(8.9)	113 698	(7.6)	46 829	(13.9)
Urban/rural						
Any MSA	1 754 859	(82.2)	1 248 215	(83.6)	260 673	(77.5)
Rural	381 277	(17.8)	245 390	(16.4)	75 761	(22.5)

Data are presented as No. (column %).

Abbreviations: AGE, acute gastroenteritis; MSA, metropolitan statistical area; PCV, pneumococcal conjugate vaccine.

a Includes children who were born in states that do not have a universal vaccination program and maintained health insurance coverage for their first 6 months.

b Definitions for all variables are included in Table 1.

c Includes both monovalent (*Current Procedural Terminology* [*CPT*] code: 90680) and pentavalent (*CPT* code: 90680) rotavirus vaccination.

Overall, 55.4% of participants (n = 1 183 658) received an antibiotic prescription during follow-up and 1.5% (n = 17 318) of those prescriptions followed an AGE diagnosis. The number of antibiotic prescriptions following an AGE diagnosis by age group and setting is presented in Supplementary Table 1. A higher proportion of children fully vaccinated against rotavirus had any antibiotic prescription during follow-up (56.7%, n = 846 883) compared to children who did not receive rotavirus vaccination (52.0%, n = 175 103). Children with full rotavirus vaccination were less likely to have an AGE diagnosis (19.7%, n = 293 655) and less likely to receive an antibiotic prescription following an AGE diagnosis (3.9% of AGE diagnoses) compared to children without rotavirus (21.2% and 4.4%, respectively).

**Figure 1.**
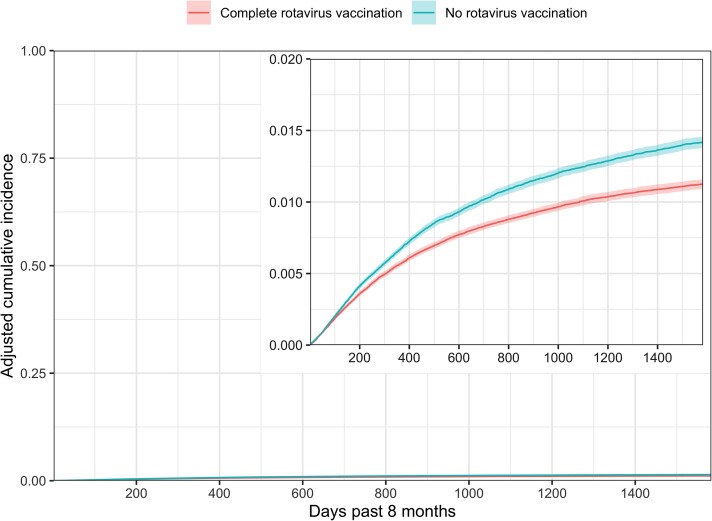
Adjusted cumulative incidence of antibiotic prescription associated with an acute gastroenteritis diagnosis, by rotavirus vaccine status, 2007–2018. Estimates are adjusted using 12 strata defined by provider type (3 categories), urban/rural (2 categories), and receipt of pneumococcal conjugate vaccine (2 categories).

**Figure 2.**
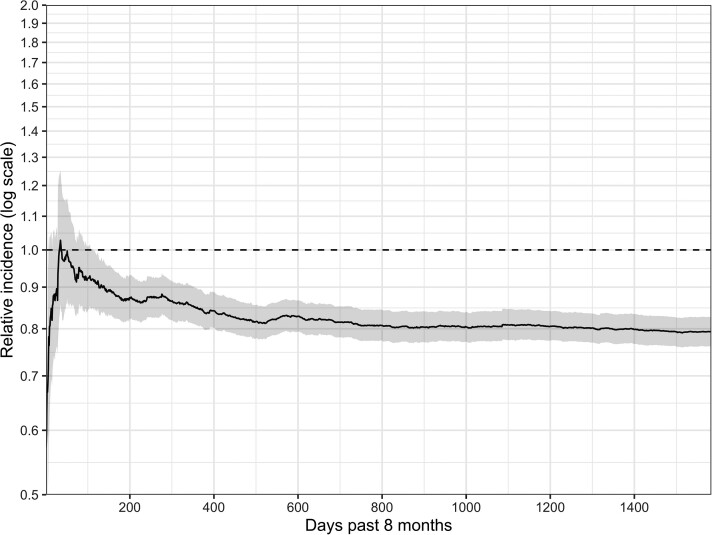
Adjusted relative incidence of antibiotic prescription associated with an acute gastroenteritis diagnosis, 2007–2018. Estimates are adjusted using 12 strata defined by provider type (3 categories), urban/rural (2 categories), and receipt of pneumococcal conjugate vaccine (2 categories). Ratio compares children with complete rotavirus vaccination vs children with no rotavirus vaccination by 8 months of age. Intervals are estimated using 1000 bootstrap replications.

At 1 year of age, the adjusted relative cumulative incidence of antibiotic prescription following an AGE diagnosis was 0.909 (95% CI, .841–.986) among children with complete rotavirus vaccination compared to children without rotavirus vaccination (Table 3, Figure 1). The adjusted relative cumulative incidence decreased over time to 0.821 (95% CI, .784–.862) at 2 years of age and 0.793 (95% CI, .761–.827) at 5 years of age (Figure 2). We report the cumulative incidence of antibiotic prescription following AGE by age group and rotavirus vaccination in Supplementary Table 2. This association was stronger when we limited the outcome to only antibiotic prescriptions following AGE diagnoses that occurred during the typical rotavirus season, demonstrated by an adjusted relative cumulative incidence ratio of 0.836 (95% CI, .762–.921) at 1 year of age and 0.729 (95% CI, .691–.773) at 5 years of age. As a sensitivity analysis, we included *ICD* codes for vomiting, and the results followed a similar pattern (Supplementary Table 3). Additionally, at 5 years of age, the adjusted relative cumulative incidence of receiving a second, unique prescription within 28 days was 0.820 (95% CI, .750–.905) among children with complete rotavirus vaccination compared to children without rotavirus vaccination.

**Table 3.** **Adjusted** ^a^  **Relative Cumulative Incidence of Antibiotic Prescription Associated With an Acute Gastroenteritis Diagnosis, at Different Ages, 2007–2018**

Age	Antibiotic Prescription Associated With AGE	Antibiotic Prescription Associated With AGE (January–June Only)	Switch of Antibiotic Prescription Associated With AGE	Any Antibiotic Prescription
	Ratio^b^ (95% CI^c^)	Ratio^b^ (95% CI^c^)	Ratio^b^ (95% CI^c^)	Ratio^b^ (95% CI^c^)
1 year	0.909 (.841–.986)	0.836 (.762–.921)	0.893 (.758–1.050)	1.050 (1.043–1.055)
2 year	0.821 (.784–.862)	0.746 (.703–.798)	0.826 (.748–.925)	1.046 (1.041–1.050)
3 year	0.806 (.771–.842)	0.735 (.697–.781)	0.806 (.736–.890)	1.039 (1.036–1.043)
4 year	0.805 (.774–.841)	0.730 (.693–.776)	0.817 (.747–.903)	1.031 (1.028–1.035)
5 year	0.793 (.761–.827)	0.729 (.691–.773)	0.821 (.750–.905)	1.025 (1.022–1.028)

Abbreviations: AGE, acute gastroenteritis; CI, confidence interval.

a Adjusted using 12 strata defined by provider type (3 categories), urban/rural (2 categories), and receipt of pneumococcal conjugate vaccine (2 categories).

b Ratio compares children with complete rotavirus vaccination vs children with no rotavirus vaccination by 8 months of age.

c Intervals are estimated using 1000 bootstrap replications.

According to vital statistics data, from 2007 to 2018 there were 48 092 250 children born in the US and based on annual trends in rotavirus vaccination coverage, we estimated that 33 150 763 (68.9%) of them were vaccinated against rotavirus. Extending our results to this cohort of children, we estimated that rotavirus vaccination has averted the prescription of at least 67 045 (95% CI, 53 729–80 664) antibiotics associated with an AGE diagnosis among children born between 2007 and 2018.

## DISCUSSION

The IBM MarketScan Commercial Database provided a unique opportunity to investigate the relationship between rotavirus vaccination and antibiotic prescriptions, which has not been previously described in the US. We found that children with rotavirus vaccination were less likely to be prescribed an antibiotic following an AGE diagnosis and were then less likely to switch antibiotics within 28 days of the initial prescription. This differential in antibiotic prescribing between fully vaccinated and unvaccinated children increased through follow-up to 5 years of age. When applied to the US child population, we estimate that rotavirus vaccination has prevented >67 000 initial antibiotic prescriptions since vaccine introduction. These findings highlight an important nontargeted benefit of rotavirus vaccination: a reduction in antibiotic prescribing among children with AGE.

We found that the protection against antibiotics following an AGE diagnosis increased over time, with the rotavirus vaccine cumulative effectiveness increasing from 9.1% (95% CI, 1.4%–15.9%) at 1 year of age to 20.7% (95% CI, 17.3%–23.9%) by 5 years of age. The instantaneous vaccine effectiveness over time is presented in Supplementary Figure 1 and indicates that vaccine effectiveness increases steeply in the first couple of months after vaccine administration and gradually increases over the following year. Furthermore, limiting our analysis to AGE diagnoses that occurred during the typical rotavirus season (January–June) resulted in a higher cumulative vaccine effectiveness of 16.4% at 1 year (95% CI, 7.9%–23.8%) and 27.1% (95% CI, 22.7%–30.9%) at 5 years, supporting a causal relationship. These results are in comparison to the estimated reduction of 11.4% within the first 2 years of life in low- and middle-income countries (LMICs) described in Lewnard et al [34]. However, it is important to note differences in treatment practices in LMICs compared to the US. Rogawski et al reported that 45.8% of diarrhea episodes within the first 2 years of life in 8 LMICs were treated with antibiotics [35], indicating that the absolute incidence of antibiotic prescribing associated with diarrhea that is averted by rotavirus vaccination is probably much higher in these settings. An additional study by Lewnard et al found that rotavirus was the leading etiologic cause of antibiotic-treated diarrhea in the first 2 years of life in LMICs and conclude that vaccination programs could substantially reduce antibiotic consumption in LMICs [21].

These results further support the utility of vaccination as part of a broader, comprehensive effort to reduce antimicrobial resistance [16]. In addition to promoting the development of new vaccines to prevent infections and the general overuse of antibiotics, the National Vaccine Advisory Committee recently highlighted the importance of existing vaccines as a tool to prevent prescribing of antibiotics for viral gastroenteritis [36]. Rotavirus vaccine coverage in the US has remained low compared to other routine immunizations. In 2016–2017, only three-quarters (75.3%) of children had completed the full rotavirus vaccine series by 8 months of age, whereas >90% of children had completed 3 doses of diphtheria, tetanus, and acellular pertussis vaccine (93.4%) or PCV (91.5%) by 2 years of age [25]. Our results suggest that the potential benefits of achieving increased vaccination coverage will extend beyond reducing rotavirus gastroenteritis to antibiotic prescribing and perhaps other outcomes.

These results should be interpreted within the context of a few limitations. First, we were unable to control for socioeconomic factors (race, ethnicity, income) that may confound our exposure-outcome relationship because they were unavailable in the database. However, controlling for PCV vaccination, provider type, and urban/rural status likely reduced the potential confounding due to known confounders [25–28]. The higher general antibiotic prescription rates among children who were vaccinated are likely a reflection of a difference in healthcare-seeking behavior between the 2 groups, but focusing our outcome on antibiotic prescriptions following an acute gastroenteritis diagnosis reduces that concern [37]. Additionally, it is possible the provider type for rotavirus vaccine administration and AGE visits may be different, which could allow for residual confounding. Second, we did not evaluate other diagnostic codes concomitant with AGE diagnostic codes, which could result in some misclassification of the outcome. This approach could lead to the inclusion of AGE diagnoses associated with other health conditions that are unrelated to vaccination, which yields a conservative estimate of effect. Similarly, if individuals did not have prescription coverage as part of their insurance at time of AGE visit, we may be underestimating prescriptions following an AGE diagnosis, which would likewise result in a conservative estimate of effect. Third, although we included antibiotic prescriptions that occurred shortly after discharge from inpatient visits with an AGE diagnostic code, we are unable to capture antibiotics that are administered during inpatient settings. Fourth, we used an insurance claims database in which low-income populations, particularly populations that are more likely to be underinsured or on Medicaid, are underrepresented or not included. Excluding these populations, among whom antibiotic prescribing is high [38, 39], could underestimate the impact of rotavirus vaccination. Finally, our results may further underestimate the impact of rotavirus vaccination because we did not specifically investigate indirect vaccine effects. Rotavirus vaccination has been shown to provide indirect (herd) protection against rotavirus gastroenteritis among both unvaccinated children and adults in the US [6]. We are unable to account for these indirect effects among the unvaccinated older populations, including adults in our study, potentially underestimating the overall effect of rotavirus vaccination. As such, we suspect that our study provides a conservative estimate of the population-level impact of rotavirus vaccination on AGE in children, and the indirect benefit among adults associated with antibiotic prescribing, and that the true impact may in fact be larger. Previous studies suggest that approximately 21–24 million antibiotics are prescribed annually for children <5 years of age in the outpatient setting [17, 38]. Although the 67 000 treated episodes averted by rotavirus vaccination according to these results are a relatively small portion of all antibiotics prescribed in this age group, these results indicate that rotavirus vaccination can be part of a multifaced approach to reducing antibiotic treatment.

Our analysis used a large, longitudinal, individual-level insurance claims database, which uniquely allowed for the detection and precise estimation of the association between rotavirus vaccination and antibiotic prescribing. These results demonstrate an additional important, nontargeted benefit of rotavirus vaccination and bolster evidence for the use of rotavirus vaccines for reducing antibiotic prescribing for acute gastroenteritis. In addition to the existing evidence that vaccination against respiratory infections may reduce antibiotic prescriptions [13], these results provide similar evidence for nonrespiratory infections as well. The reduction of antibiotic prescribing likely contributes to the broader effort of reducing antimicrobial resistance. Thus, increasing rotavirus vaccination coverage should be encouraged both for its intended and nontargeted effects.

## Supplementary Material

ofac276_Supplementary_Data

## Supplementary Data

Supplementary materials are available at *Open Forum Infectious Diseases* online. Consisting of data provided by the authors to benefit the reader, the posted materials are not copyedited and are the sole responsibility of the authors, so questions or comments should be addressed to the corresponding author.

## Notes

***Patient consent.*** As the MarketScan database is compliant with the Health Insurance Portability and Accountability Act, no additional ethics committee approval was needed for this research. Patient consent was not needed for this study.

***Financial support.*** This work was supported by funding from the Wellcome Trust (grant number 219823/Z/19/Z) and the National Institute of Allergy and Infectious Diseases (grant number T32AI074492).

***Potential conflicts of interest.*** B. L. reports grants and personal fees from Takeda Pharmaceuticals and personal fees from the World Health Organization (WHO), unrelated to the submitted work. E. W. H. reports consulting fees from Merck, unrelated to the submitted work. J. M. B. reports consulting fees from WHO, unrelated to the submitted work. All other authors report no potential conflicts of interest.
