# Supplementary material for: Association Between Rotavirus Vaccination and Antibiotic Prescribing Among Commercially Insured US Children, 2007–2018
Source: Open Forum Infect Dis. 2022 Jun 9;9(7):ofac276. doi: 10.1093/ofid/ofac276 (PMC9291383; doi:10.1093/ofid/ofac276)
Supplement: ofac276_Supplementary_Data [file ofac276_supplementary_data.zip › Supplementary Data 5_2_22.docx]

**Title:** Association Between Rotavirus Vaccination and Antibiotic Prescribing among Commercially Insured US Children, 2007-2018

**Authors:** Eric W. Hall^1^, PhD, Ashley Tippett^2^, MPH, Scott Fridkin^3^, MD, Evan J. Anderson^2,3,4^, MD, Ben Lopman^5^, PhD, David Benkeser^6^, PhD*, Julia M Baker^5^, PhD*

*Contributed equally

**Affiliations:**

^1^School of Public Health, Oregon Health & Science University, Portland, OR, USA

^2^Department of Pediatrics, Emory University School of Medicine, Atlanta, GA, USA

^3^Department of Medicine, Emory University School of Medicine, Atlanta, GA, USA

^4^Center for Childhood Infections and Vaccines, Children’s Healthcare of Atlanta, Emory University, Atlanta, GA, USA

^5^Department of Epidemiology, Rollins School of Public Health, Emory University, Atlanta, GA

^6^Department of Biostatistics and Bioinformatics, Rollins School of Public Health, Emory University, Atlanta, GA, USA

**Supplementary Data**

Supplementary Table 1. Number of participants with acute gastroenteritis (AGE) and antibiotic prescription associated with AGE, by age of first occurrence and setting, 2007-2018.

|  | Overall | | |  | Outpatient | | |  | Inpatient | | |  |
| --- | --- | --- | --- | --- | --- | --- | --- | --- | --- | --- | --- | --- |
| **Age** | **First AGE**^a^ **(n)** | **With antibiotic**^a^ **(n)** | **%** |  | **First AGE**^a^ **(n)** | **With antibiotic**^a^ **(n)** | **%** |  | **First AGE**^a^ **(n)** | **With antibiotic**^a^ **(n)** | **%** | |
| <1 year | 141,893 | 10,773 | 7.6 |  | 140,396 | 10,202 | 7.3 |  | 1,497 | 571 | 38.1 | |
| 1-2 years | 196,420 | 3,895 | 2.0 |  | 194,589 | 3,742 | 1.9 |  | 1,831 | 153 | 8.4 | |
| 2-3 years | 57,798 | 1,625 | 2.8 |  | 57,277 | 1,553 | 2.7 |  | 521 | 72 | 13.8 | |
| 3-4 years | 21,726 | 823 | 3.8 |  | 21,510 | 787 | 3.7 |  | 216 | 36 | 16.7 | |
| 4-5 years | 10,275 | 202 | 2.0 |  | 10,155 | 191 | 1.9 |  | 120 | 11 | 9.2 | |
| Overall | 428,112 | 17,318 | 4.0 |  | 423,927 | 16,475 | 3.9 |  | 4,185 | 843 | 20.1 | |

^a^Full definitions are included in Table 1.

Supplementary Table 2. Adjusted^a^ cumulative incidence of antibiotic prescription associated with an acute gastroenteritis diagnosis, by different ages, 2007-2018.

|  | Complete rotavirus vaccination^b^ | | |  | No rotavirus vaccination | | |  | Difference | | |
| --- | --- | --- | --- | --- | --- | --- | --- | --- | --- | --- | --- |
| **By age** | **median** | **lower** | **upper** |  | **median** | **lower** | **upper** |  | **median** | **lower** | **upper** |
| 1 year | 0.224% | 0.212% | 0.239% |  | 0.247% | 0.234% | 0.261% |  | 0.023% | 0.003% | 0.041% |
| 2 year | 0.685% | 0.663% | 0.711% |  | 0.835% | 0.807% | 0.861% |  | 0.150% | 0.113% | 0.184% |
| 3 year | 0.901% | 0.875% | 0.929% |  | 1.119% | 1.084% | 1.150% |  | 0.217% | 0.174% | 0.261% |
| 4 year | 1.042% | 1.011% | 1.075% |  | 1.295% | 1.259% | 1.330% |  | 0.253% | 0.204% | 0.299% |
| 5 year | 1.124% | 1.091% | 1.158% |  | 1.417% | 1.377% | 1.456% |  | 0.292% | 0.239% | 0.346% |

^a^Adjusted using 12 strata defined by provider type (3) x urban/rural (2) x PCV vaccination (2)

^b^Includes both monovalent (CPT code: 90680) and pentavalent (CPT code: 90680) rotavirus vaccination.

Supplementary Table 3. Adjusted^a^ relative cumulative incidence of antibiotic prescription associated with an acute gastroenteritis diagnosis, by definition of acute gastroenteritis, 2007-2018.

|  | Antibiotic prescription associated with AGE  (primary results)^b^ | Antibiotic prescription associated with AGE  (including vomiting)^c^ |
| --- | --- | --- |
| **By age** | **Ratio^d^ (95% Ci^e^)** | **Ratio^d^ (95% Ci^e^)** |
| 1 year | 0.909 (0.841, 0.986) | 0.931 (0.880, 0.997) |
| 2 year | 0.821 (0.784, 0.862) | 0.866 (0.835, 0.900) |
| 3 year | 0.806 (0.771, 0.842) | 0.856 (0.829, 0.887) |
| 4 year | 0.805 (0.774, 0.841) | 0.853 (0.826, 0.881) |
| 5 year | 0.793 (0.761, 0.827) | 0.835 (0.809, 0.862) |

^a^Adjusted using 12 strata defined by provider type (3) x urban/rural (2) x PCV vaccination (2)

^b^Includes ICD-9/ICD=10 codes reported in Table 1.

^c^Includes ICD-9/ICD=10 codes reported in Table 1 plus the following codes for vomiting: 787.03, 787.01 (ICD-9) and R11.1, R11.10-R11.12, R11.2 (ICD-10).

^d^Ratio compares children with complete rotavirus vaccination vs children with no rotavirus vaccination by 8 months of age.

^e^Intervals are estimated using 1000 bootstrap replications.

Supplementary Figure 1. Adjusted^a^ instantaneous vaccine effectiveness of rotavirus vaccination on antibiotic prescription associated with an acute gastroenteritis diagnosis, 2007-2018.

^a^Adjusted using 12 strata defined by provider type (3) x urban/rural (2) x PCV vaccination (2)
