# Supplementary figures and images for: Association Between Rotavirus Vaccination and Antibiotic Prescribing Among Commercially Insured US Children, 2007–2018
Source: Open Forum Infect Dis. 2022 Jun 9;9(7):ofac276. doi: 10.1093/ofid/ofac276 (PMC9291383; doi:10.1093/ofid/ofac276)

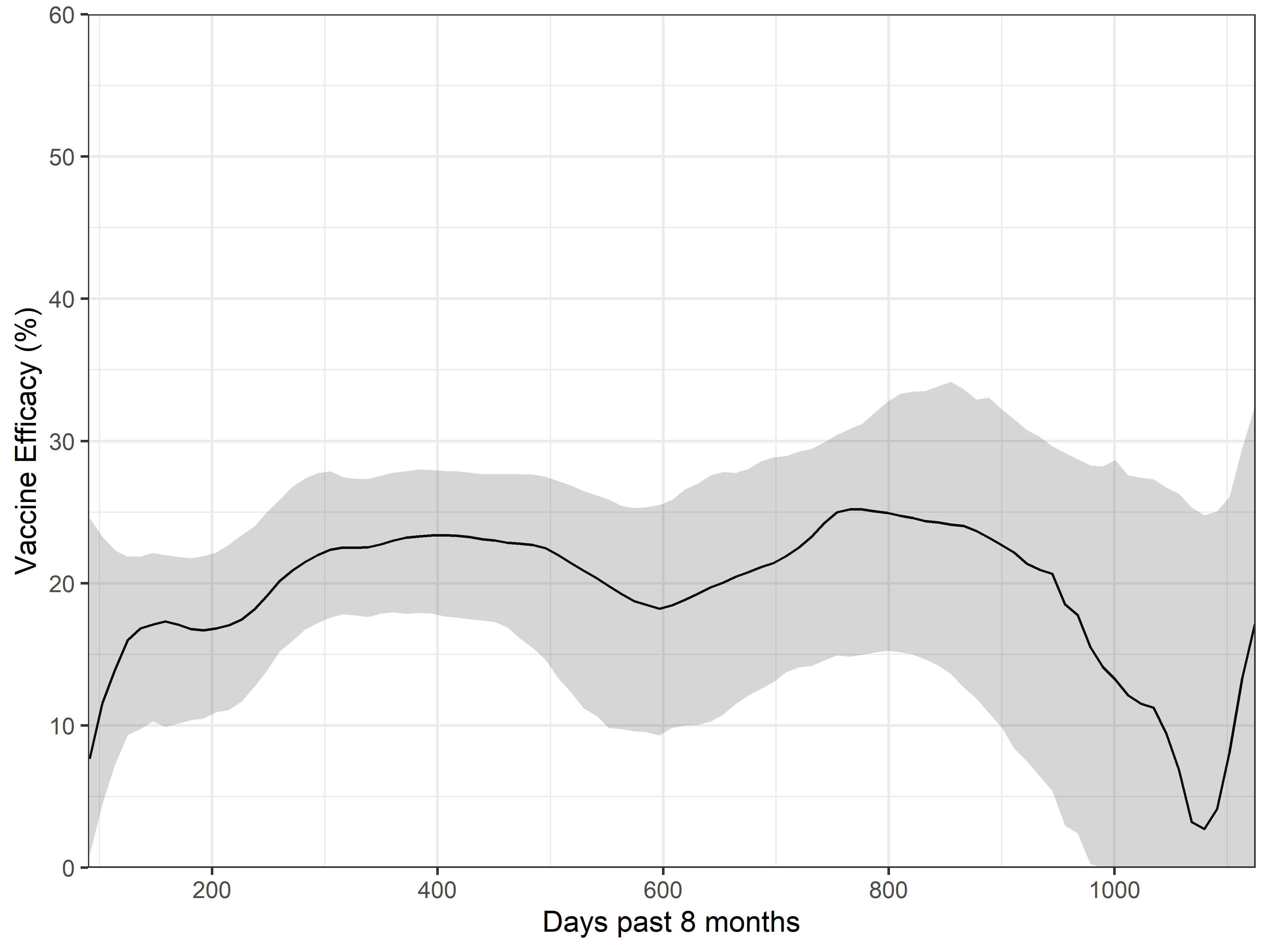

Supplement: ofac276_Supplementary_Data [file ofac276_supplementary_data.zip › Supplementary Figure 1.png]
